# COVID-19’s effects on the Egyptian population’s brain: Could MRI and specialized MR spectroscopic analysis be beneficial?

**DOI:** 10.1186/s43055-022-00911-z

**Published:** 2022-10-20

**Authors:** Alaa Mohamed Reda, Ahmed Elsharkawy, Tamer Mahmoud Dawoud

**Affiliations:** Faculty of Medicine, Tanta University, El-Geish Street, Tanta, Gharbia Governorate Egypt

**Keywords:** Neurological manifestation of COVID-19, MRI brain, MRS in COVID-19’s patients

## Abstract

**Background:**

The recent pandemic of COVID‐19 has thrown the world into chaos due to its high rate of transmissions. Recently viewed neurological manifestations among hospitalized Egyptian patients with COVID‐19 in quarantine centres. Ataxia, disturbed consciousness and convulsions should be further evaluated by MRI and MRS for CNS involvement by SARS‐CoV‐2. How COVID-19 targeting the CNS is still under study, as it is difficult to predict which diagnostic neurological tests will be used to identify high-risk COVID19 patients. MR spectroscopy represents a non-invasive in vivo diagnostic technique for evaluation of metabolic profile of the brain and can reveal important information about the underlying pathologies. Multiple recent reports in the medical literature had confirmed the neurological complications in COVID-19 infection, though few studies has reported the MR spectroscopic findings in these patients. This cross-sectional study aimed to use MRI and MR spectroscopic findings for evaluation of the neurological manifestation of Egyptian COVID‐19 patients.

**Results:**

Ninety-one male and twenty-seven female met the inclusion criteria, with a mean age of 52 years ± 10 (SD) (age range; 12–78 years). The commonest neurological manifestations were disturbed conscious level (82.2%). The most common MRI findings were acute ischemic insult with/without haemorrhagic areas (42.3%), demyelinating patches of altered signal intensity (31.3%). Sixty cases who had haemorrhagic areas were excluded to perform MRS due to contamination of the spectra by blood component. However, the remaining 67 patients had NAA reduction, choline elevation, glutamate/glutamine and lactate elevation in short TE35, with mean of NAA/Cr ratio = 1.04 ± 0.14, Choline/Cr = 0.49 ± 0.04 and Glx/Cr = 1.56 ± 0.22.

**Conclusions:**

During the current pandemic of COVID-19, radiologists should be aware of wide spectrum of MRI and MRS findings of COVID-19-related CNS involvement.

## Background

Severe acute respiratory syndrome coronavirus 2 (SARSCoV-2) is the seventh member of the family of coronaviruses that infects people and causes infection with COVID19 [1]. Corona viruses can invade Central Nervous System (CNS) by two mechanisms: viral replication into glial or neuronal cells of the brain or autoimmune reaction [2]. In the last 2 years, many cases of acute encephalitis caused by human coronaviruses have been identified [3]. Regarding to the current pandemic of COVID-19, central nervous system involvement is still uncommon but growing in their number and demonstrating a higher frequency of neurological signs and symptoms as motor weakness, delirium, aphasia, sensory disorders, loss of consciousness, seizures, coma [4].

MR spectroscopy represents a non-invasive in vivo diagnostic technique for evaluation of metabolic profile of the brain and can provide valuable information about the underlying pathologies. It offers a complementary method to the structural conventional MRI data by measuring the different neuronal metabolites. Furthermore, even in the absence of conventional MRI structural abnormalities, it may provide valuable information about neurochemical abnormalities [5].

Multiple recent reports in the medical literature had confirmed the neurological complications in COVID-19 infection, though few studies has reported the MR spectroscopic findings in these patients. This cross-sectional study aimed to use MRI and MR spectroscopic findings for evaluation of the neurological manifestation of Egyptian COVID‐19 patients.

## Methods

This cross-sectional cohort study was performed from May 23, 2020, to January 15, 2022 in two large quarantine centres. The study was accepted by our institute's ethical committee of our institute. Written informed consent from patients or their relatives after assurance of benefits of the study.

### Inclusion criteria

One hundred twenty-seven patients of positively RT-PCR test who had neurological signs and symptoms as motor weakness, delirium, aphasia, sensory disorders, loss of consciousness, seizures, coma (that appeared after COVID-19 exposure), with no previous neurological or psychiatric disorders were included in this study.

### Exclusion criteria

**Fig. 1:**
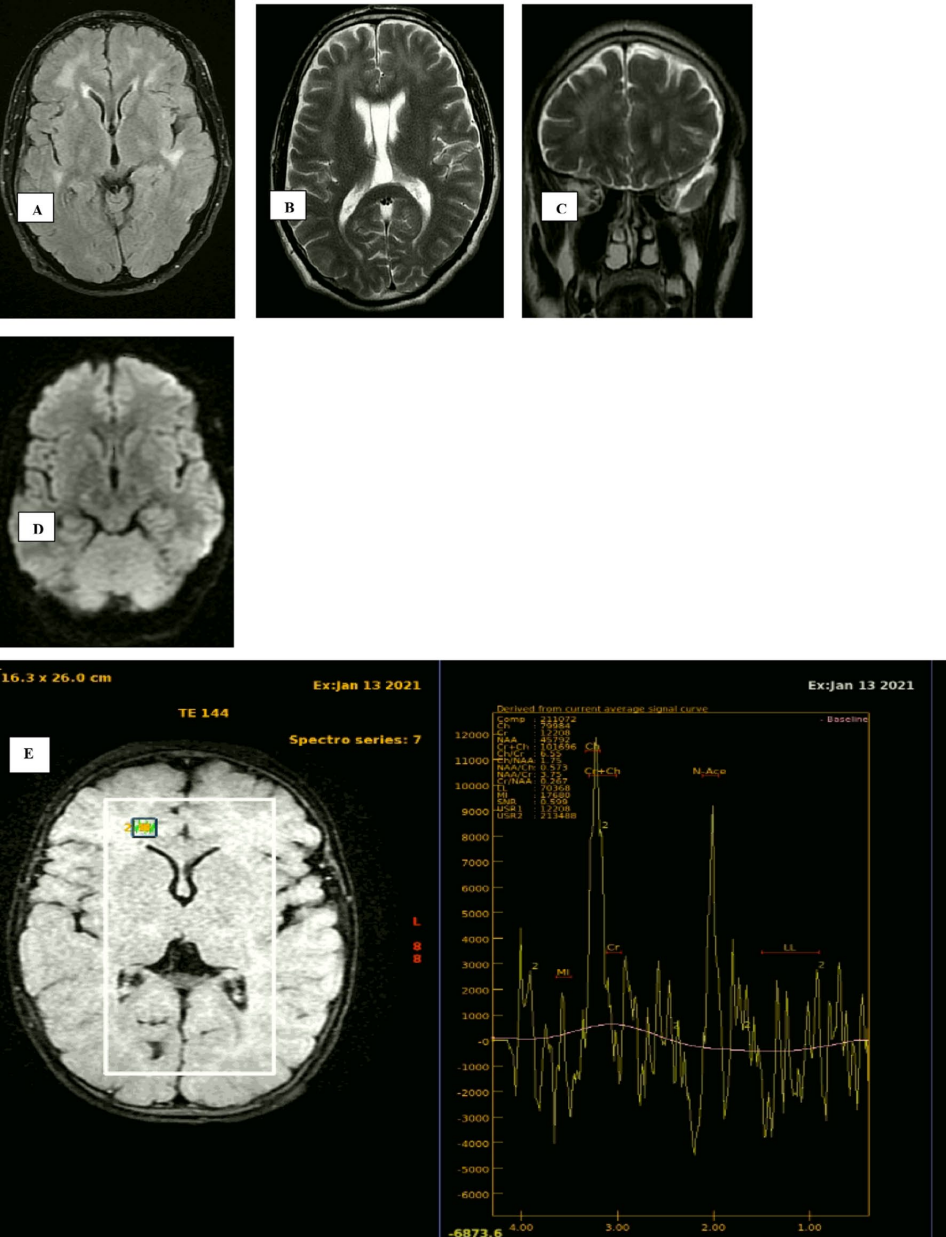

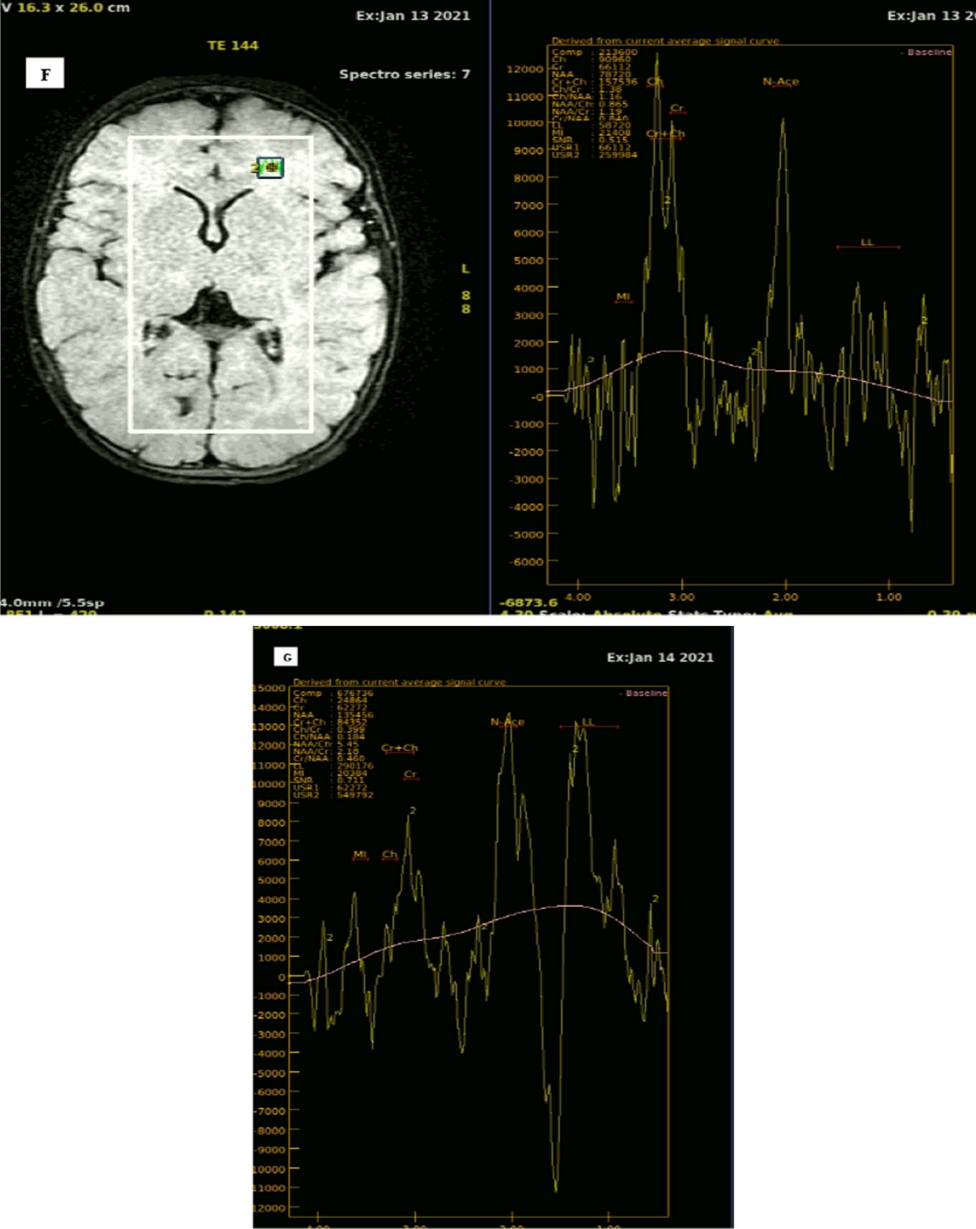
A 49-year-old man with positive PCR for COVID-19 presented with fever, dyspnoea, after 10 days the patient came with convulsions. **A** Axial FLAIR showing non-confluent multifocal white matter hyper intense lesions are seen in both fronto-temporal lobes and periventricular regions, **B** axial T2WI, showing hyperintense signal of the previously mentioned areas of altered signal, **C** coronal T2WI, **D** DWI (*b*-value 1000) showing free diffusion of the areas of altered signal, **E** long TE 144 (at right frontal region), **F** long TE 144 (at left frontal region); both show mild increase level of choline with mild decrease in NAA peak, **G** short TE35 show high bifid peak of lipids and lactate…. Diagnosed as COVID induced demyelination

Patients who had contraindications to undergo MRI (as metallic prosthesis, cardiac pacemakers, intraocular metallic foreign bodies) as well as claustrophobic patients were excluded from the study. Patients who suffered from previous neuropsychiatric disorders or even systemic diseases that manifested with neurological symptoms as previous stroke, moto weakness, sensory disorders, aphasia, seizures, coma and/or previous brain MRI changes (Fig. 1).

## Brain MRI protocols

Images acquisition was conducted on a 1.5-Tesla MRI machine. The used MRI sequences were T1-weighted spin-echo MRI with or without Gadolinium contrast injection (TR 400–600, TE 15–25), T2-weighted spin-echo (TR 3000–4000, TE 100–120), diffusion-weighted imaging (B-value; 0, 1000), susceptibility-weighted imaging, and two-dimensional fluid-attenuated inversion recovery (FLAIR) sequence (TR 7.000–9000, TE 110).

Multi-voxels MR spectroscopy (MRS) was performed in 65 patients (55%) with intermediate TE at 144 ms and short TE 35 ms after shimming (to adjust proper field homogeneity) and assessment of bandwidth below 10. MRS volume was placed over the anatomical region of interest with caution not to include fat-containing scalp or bone marrow that may contaminate the spectrum. Outer saturation bands were placed to supress signal from fat.

**Fig. 2:**
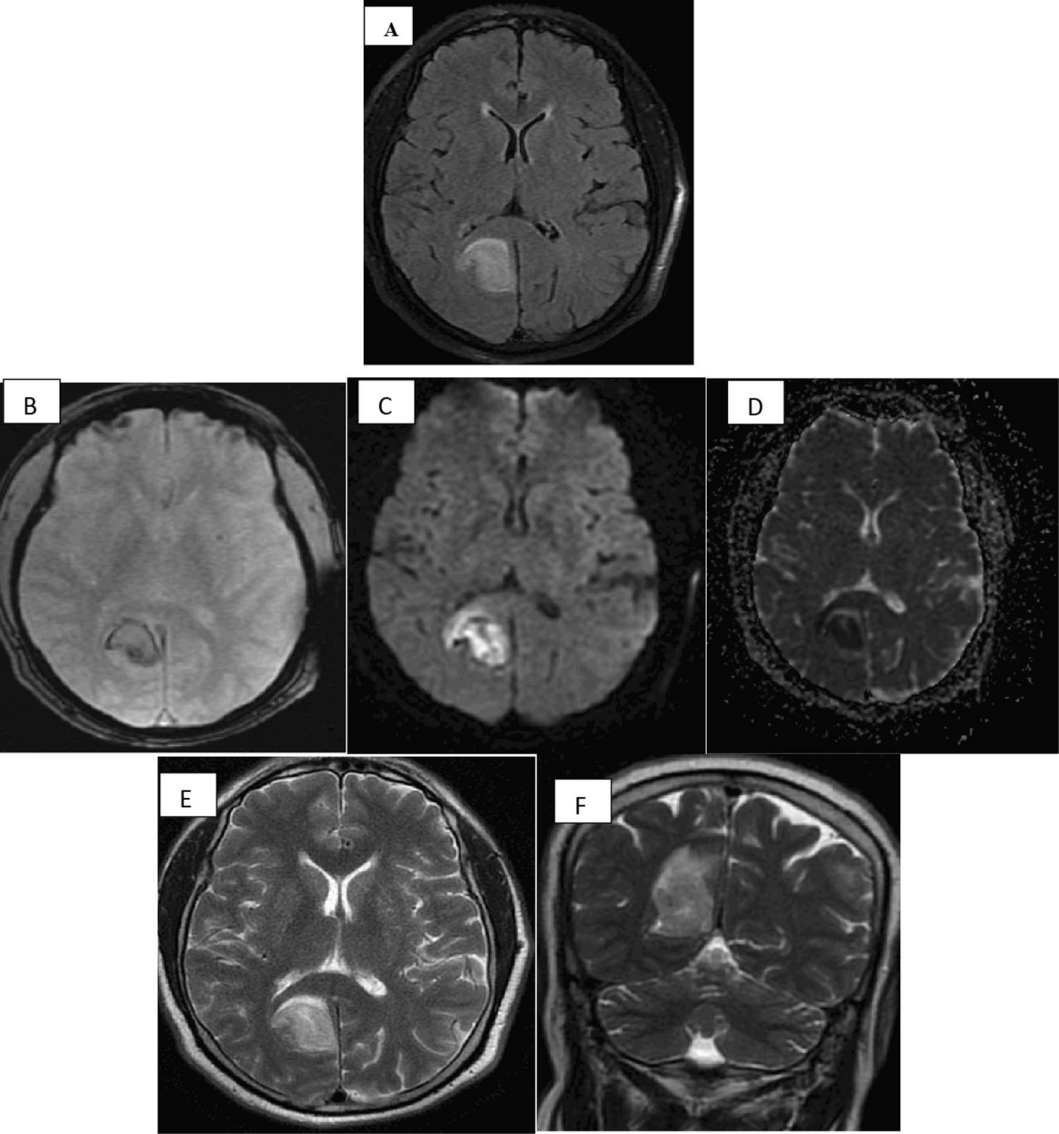
A 58-year-old female with COVID-19 infection confirmed by PCR test, presented with fever, cough, 1 week later, she came with unilateral weakness. Cortical and sub-cortical area of altered signal intensity is seen in the right occipital lobe. **A** Axial FLAIR showing right occipital area of heterogenous signal intensity. **B** Axial gradient sequence showing right occipital area of heterogenous signal intensity with rim of low signal intensity. **C** DWI (*b*-value 1000) showing restricted diffusion. **D** ADC image, showing suppression of restricted high diffusion signal. **E** Axial T2WI, **F** coronal T2WI showing heterogenous signal intensity. Diagnosis: Right posterior cerebral arterial territory recent haemorrhagic infarction

MRS spectra datasets were analyzed visually and also quantitatively to identify and quantify ratios of NAA, Cho, mI, and Glx relative to Cr. Lactate (Lac)/Cr levels were determined using MRS datasets with long TEs, Table 4 summarises the means, SDs and ranges of metabolite levels (Fig. 2).

## MRI interpretation

After anonymization, images were presented to readers on a picture archiving and communication system (General Electric, Toshiba).

**Fig 3:**
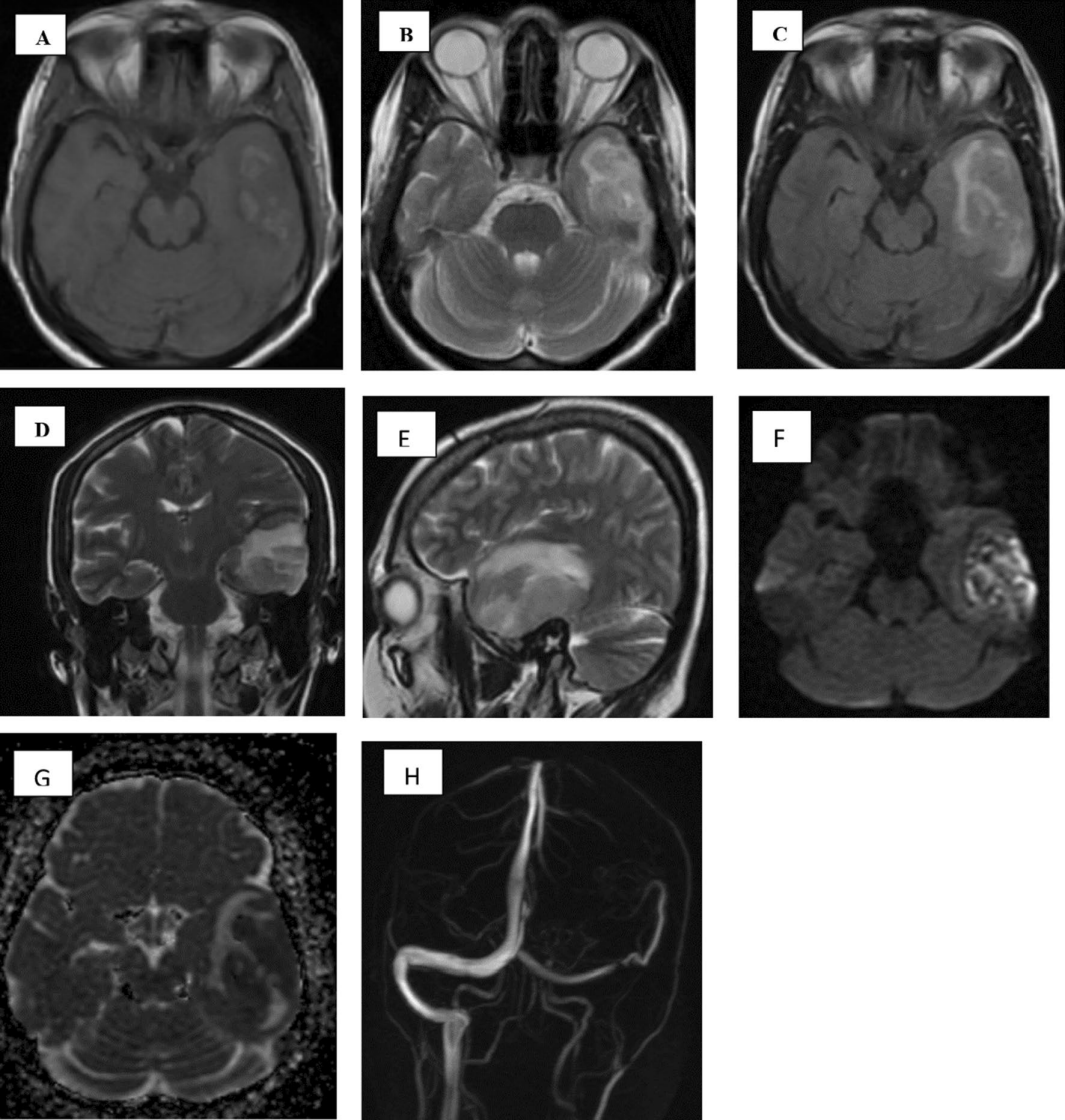
A 63 years-old infected man with COVID-19, presented by blurred vision and vomiting 14 days after initial symptoms, high D-dimer level, MRI study was done and showing left temporal area of altered signal intensity, **A** axial T1WI shows areas of hyperintensities, **B** axial T2WI shows heterogenous signal intensities, **C** axial FLAIR sequence displays high signal, **D** coronal T2WI, **E** sagittal T2WI, **F** DWI has areas of restricted diffusion, **G** ADC image, MRV in (**H**, image), shows marked attenuation in left sigmoid, transverse sinus with focal area of signal loss in left transverse sinus and non-visualized left IJV, this is consistent with sinus thrombosis and left temporal haemorrhagic infarction

Post-processing of MRS was performed using MRI workstation software (ADW 4.7 Vantage, GE Medical Systems) by dedicated software, values of brain metabolites were assessed by placing small Regions of Interest (ROIs) that were manually drawn, measuring 2 × 2 mm^3^ in the areas of altered signal intensity. After review of MRI studies by the study authors who are two experienced radiologists of about 8 years’ experience in neuroradiology, their findings were analysed. The final decision was done by a third neuro-radiologist with 11 years of experience in neuroradiology field if there were disagreements between the formers’ opinions. Each patient took a different ID number, and all patient data were blinded (Fig. 3).

## Statistical tests used

Categorial variables were described by frequency and proportion and quantitative variables were described by using mean, SD and range. Categorical data were compared using the Fisher exact test, meanwhile quantitative data were compared by using the student *t* test. *P* value less than 0.05 represented a significant difference.

## Results

Between May 23, 2020, to January 15, 2022, the current study was conducted on 127 positive RT-PCR of SARS-COV-2 who were hospitalized in a large quarantine centre. Nine patients who had contraindications to undergo MRI (as metallic prosthesis) were excluded from the study, while 118 cases met the inclusion criteria. Clinical data and laboratory findings were evaluated and tabulated. Ninety-one males (77.2%) and twenty-seven females (22.8%) met the inclusion criteria, with a mean age of 52 years ± 10 (standard deviation) (age range, 12–78 years), as shown in (Table 1). The commonest neurological manifestations were disturbed conscious level (82.2%), confusion (31.3%), agitation (16.1%), seizures (14.4%) and coma (11%). The mortality rate at the end of the study was 12.7%. The laboratory findings of patients showed leucocytosis, lymphopenia, elevated serum levels of C-reactive protein, ferritin, urea, creatinine, fibrinogen, and d-dimers, as shown in (Table 2).

**Table 1:** Demographic parameters and clinical characteristics in the studied cases

Demographic and clinical parameters	All patients (*n* = 118)	Non-haemorrhagic forms (*n* = 63)	Haemorrhagic forms (*n* = 55)	*P* value
Sex*				
Male	91 (77.2%)	51 (80.9%)	40 (72.7%)	0.38
Female	27 (22.8%)	12 (19%)	15 (27.2%)	
Age (years)*	Mean age of 52 ± 10 (12–78)	Mean age = 58 (12–71)	Mean age of 65 (49–78)	0.13
Time interval from onset of respiratory symptoms to first hospital admission, mean-range*	14 d (0–28)	12 d (8–28)	8 d (0–12)	0.81
Time interval from onset of neurological symptoms to brain MRI (d) mean-range*	30 h (4 h–3d)	48 h (36 h–3d)	8 h (4–12 h)	0.01
Admission to ICU*	101 (85.5%)	47 (75%)	54 (98%)	0.02
Respiratory distress syndrome*	97 (82.2%)	42 (66.7%)	55 (100%)	0.04
Oxygen therapy*	115 (97.4%)	60 (95.2%)	55 (100%)	0.47
Past medical history*				
History of stroke	24 (20.3%)	6 (9.5%)	18 (32.7%)	0.08
History of seizures	6 (5%)	6 (9.5%)	0	0.48
Another neurologic diseases	57 (48.3%)	35 (55.5%)	22 (40%)	0.42
No previous neurological disorders	31 (26.2%)	8 (12.6%)	23 (41.8%)	0.47
Death*	15 (12.7%)	4 (6.3%)	11 (20%)	0.37
Clinical manifestations*				
Disturbed conscious level	97 (82.2%)	45 (71.4%)	52 (94.5%)	0.14
Confusion	37 (31.3%)	24 (38%)	13 (23.6%)	0.17
Agitation	19 (16.1%)	12 (19%)	7 (12.7%)	0.31
Seizures	17 (14.4%)	7 (11.1%)	10 (18.8%)	0.94
Headache	14 (11.8%)	10 (15.8%)	4 (7.2%)	0.30
Coma	13 (11%)	9 (14.2%)	4 (7.2%)	0.62

*Data are numbers of patients, and data in parentheses are percentages

*Data are means, with ranges in parentheses and medians in brackets

*P value less than 0.05 indicates a significant difference

**Table 2:** Laboratory findings in the studied cases

Laboratory findings	All patients (Mean, range)	Non-haemorrhagic forms (*n* = 63) (No., mean, range)	Haemorrhagic forms (*n* = 55) (No., mean, range)	*P* value
WBCs count (10^9^ × L) (*n* = 118)*	13.8 (7.6–19.2)	(63/63) 9.4 (7.6–15.7)	(55/55) 13.8 (11–19.2)	0.04
Lymphocytic count (10^9^ × L) (*n* = 118)*	1.12 (0.51–1.7)	(63/63) 1.34 (0.51–1.47)	(55/55) 1.06 (0.89–1.7)	0.9
CRP level (mg/L) (*n* = 118)*	60.5 (19–172)	(59/63) 71 (21–172)	(54/55) 44 (19–146)	0.87
Fibrinogen (g/L) (*n* = 32)*	7.3 (4.9–9.5)	(3/9) 8.4 (7.2–9.5)	(11/23) 6 (4.9–8)	0.07
D-dimer (mg/L) (*n* = 118)*	2.9 (0.7–4)	(42/63) 1.7 (0.7–3.5)	(49/55) 3.4 (2.3–4)	0.08

Data are medians and data in parentheses are the interquartile range, *n* is the number of patients, *P* value less than 0.05 indicates a significant difference. *Data are numbers of patients, and data in parentheses are percentages

Twenty-one cases underwent a lumbar puncture, CSF analysis revealed that (52.3%) of them had increased inflammatory markers (high white blood cells, high protein levels), as shown in Table 3.

**Table 3:** CSF analysis findings in the studied subjects:

CSF analysis	All patients (No., %)	*P* value
High WBCs level*	(11/21) 52.3%	0.72
High proteins level*	(11/21) 52.3%	0.97
Elevated oligoclonal IgG levels*	(8/21) 38.09%	0.01*
Positive RT-PCR SARS -COV2*	(1/21) 4.7%	0.02*
High interleukins 6 & 10 Levels*	(2/21) 9.5%	0.05*

Data are numbers of patients and data in parentheses are percentages*, n* is the number of patients.

*CSF* cerebrospinal fluid, *IgG* immunoglobulin G, *RT-PCR* reverse transcriptase polymerase chain reaction, *SARS-CoV-2* severe acute respiratory syndrome coronavirus disease 2

### The neurological findings in conventional MRI include

The most common MRI findings were acute ischemic insult with/without haemorrhagic areas (54 cases, 42.5%), acute disseminated encephalomyelitis (ADEM) (39 cases, 30.7%), encephalitis (24 cases, 18.8%) and grey matter areas of high FLAIR signal intensity either hippocampal or extra-hippocampal (4 cases, 3.1%), 3 cases of frank haemorrhage, 2 cases of microbleeds, lastly one patient of leptomeningeal affection, (as described in Table 4).

**Table 4:** MRI abnormalities in the studied cases

Brain MRI findings	No. (127)		%
Infarctions: Cortical, subcortical, single/multiple territories/watershed infractions/hemorrhagic and non-hemorrhagic	54		42.5
	Hemorrhagic 33	Non- hemorrhagic 21	
Frank hemorrhage: parenchymal, subdural, subarachnoid, single/multiple, central/peripheral	3		2.3
Microbleeds: Basal ganglia, thalami, corpus callosum, internal capsules, cerebellar peduncles seen in SWI sequence	2		1.5
Demyelinating patches as ADEM: hemorrhagic/non-hemorrhagic	39		30.7
	Hemorrhagic 17	Non-hemorrhagic 22	
Grey matter areas of altered signal: Cortical/deep nuclei Hippocampal/extra-hippocampal	4		3.1
Leptomeningeal affection: Enhancement/high signal in FLAIR, Focal (less than 3 sulci)/diffuse (more than 3 sulci)/	1		0.87
Encephalitis, multiple patchy areas of altered signal, unilateral or bilateral, not in arterial territory distribution	24		18.8
	Hemorrhagic necrotizing encephalitis 5	Non hemorrhagic 19	

**Fig. 4:**
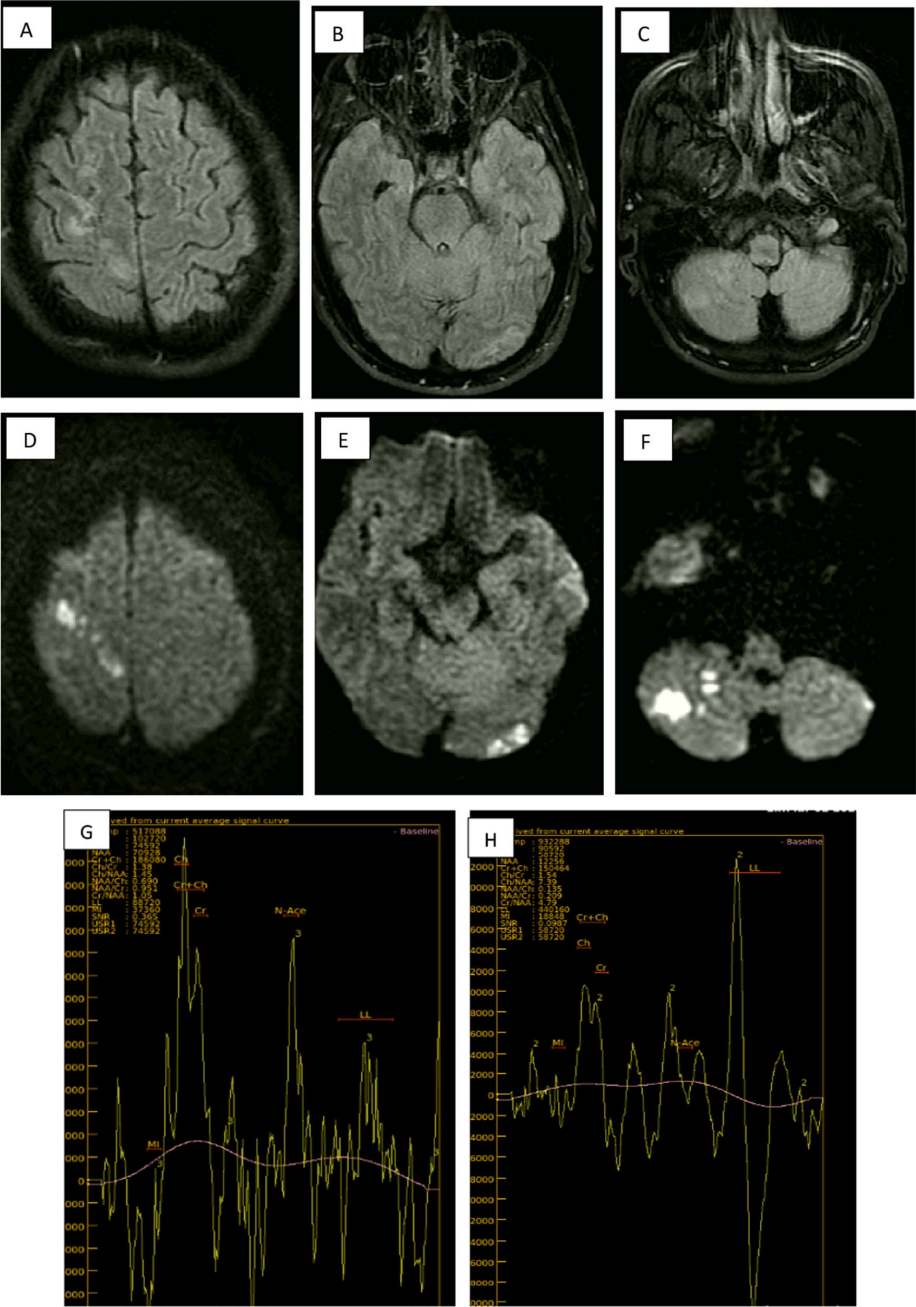
A 39-year-old male with dyspnea and fever, 6 days later, he presented with disturbed gait, weakness of both lower limbs and dysarthria. **A** Axial FLAIR sequence at high parietal regions, **B** axial FLAIR at parieto-occipital regions showing areas of altered high signal intensity, **C** axial FLAIR at both cerebellar hemispheres levels showing right cerebellar areas of altered high signal intensity. **D** Axial DWI at high parietal level, **E** axial DWI at parieto-occipital level, **F** axial DWI at both cerebellar levels showing restricted diffusion of the previously mentioned areas of altered high FLAIR signal intensity. **G** Long TE(144) of MRS, showing mild decreased NAA, creatine, myoinositol, mild elevated choline peak, **H** short TE(35) of MRS showing shooting of lipids/lactate peaks (consistent with necrosis and anaerobic metabolism). Diagnosis was multiple non-hemorrhagic infarctions. Diagnosis: multiple recent infarctions along both PCA and right posterior-inferior cerebellar artery territories)

Haemorrhagic cases (either microhaemorrhage that was detected in SWI sequences, frank hematomas or mixed with other lesions as haemorrhagic infarction, demyelinating patches or encephalitis) were represented by 60 patients, meanwhile non haemorrhagic cases were 67 patients (Fig. 4).

Comparison of the haemorrhagic versus the non-haemorrhagic cases, as follow; regarding the need for intensive care admission is more frequently associated with cases of haemorrhage than non-haemorrhagic lesions (98% vs 75% of their cases) (*P* value = 0.02), the wakefulness after stoppage of sedative treatments were noted in (64% vs 19% of their cases) (*P* value = 0.01), the time interval between the onset of the clinical neurological manifestations and brain MRI were shorter in haemorrhagic lesions (ranging from 4-12 h) versus non haemorrhagic lesions (36 h- 3 days) (*P* value = 0.01) as shown in Table 1. The laboratory changes (Table 2) were more pronounced in haemorrhagic lesions as follows; (elevated WBCs 13.8 × 10^9^/L vs 9.4 × 10^9^/L, (*P* value = 0.04), Ferritin level (mean 1299 µg/L vs 1164 µg /L, (*P* value = 0.65), Urea level (17 mmol/L vs 11 mmol/L; (*P* value = 0.26).

## MR spectroscopic metabolic profile

Fifty-five cases who were associated with haemorrhagic areas were excluded to perform MRS due to high band width (higher than 10) from contamination of the spectra by blood component. However, the remaining 63 patients underwent MRS and there was mild NAA reduction, mild choline elevation, glutamate/glutamine and lactate elevation.

Comparative analysis showed decreased NAA/Cr levels within the intra-lesional voxels rather than normal appearing white matter, NAA levels were the most diminished in cases with necrotizing encephalitis due to marked neuronal loss, followed by demyelinating changes, that was statistically significant difference (*P* value < 0.001*). Meanwhile Cho/Cr levels were indistinguishable from those at normal appearing white matter voxels, with no statistically significant difference (*P* value = 0.53). Moreover, Glx/Cr was increased in intra-lesional voxels. Lac/Cr ratios were increased in cases with necrotizing leukoencephalopathy. No elevation of Lac/Cr ratios was seen in the normal appearing white matter voxels, with statistically significant difference (*P* value = 0.027*), MRS findings were non-specific apart from decreased NAA/Cr elevated Lac/Cr ratios, and more significant changes in severe cases of necrotizing leukoencephalopathy. (Table 5: shows the metabolites profile of the studied cases).

**Table 5:** MRS metabolites analysis in the studied cases

Metabolic ratios (*n* = 63)	Intra-lesional voxels (Mean, SD and Range)	Voxels on normal appearing white matter (Mean and SD)	*P* value
NAA/Cr^a^	1.04 ± 0.14 (0.61–1.47)	1.57 ± 0.17	< 0.001*
Cho/Cr^a^	0.49 ± 0.04 (0.27–0.66)	0.28 ± 0.02	0.53
mI/Cr^a^	1.12 ± 0.05 (1.09–1.25)	0.79 ± 0.04	0.71
Glx/Cr^a^	1.56 ± 0.22 (1.1–1.83)	0.89 ± 0.07	0.92
Lc/Cr^b^	0.76 ± 0.1 (0.69–0.87)	No lipid peak	0.027*

^a^Data measured on short-TE (TE = 35 ms) spectra

^b^Data measured on Intermediate-TE (TE = 144 ms) spectra

## Discussion

The primary manifestations of COVID-19 are respiratory in nature. However, it becomes clear that SARS-CoV-2 has multi-organ affection including the central nervous system [6].

The majority of patients had MRI abnormalities with serious and various findings beyond the severe respiratory disease: cerebrovascular disease (especially ischemic stroke; large artery infarctions more frequently than watershed cerebral infarctions), venous infarction and encephalitis (including limbic encephalitis, radiologic ADEM, and radiologic acute haemorrhagic necrotizing encephalopathy). The heterogeneity of the imaging abnormalities was underpinned by heterogeneous clinical manifestations, ranging from headache to more severe findings such as confusion with agitation, disturbed consciousness level, confusion, seizures and coma [7].

In the present study, 118 patients were included. In which males were more affected than females as there were 91 males (77.2%) and 27 females (22.8%) and range of age of the enrolled 118 patients was between twelve to seventy-eight years with a mean age of 52 years, this was in agreement with Mao et al. who reported that the mean (SD) age was 52.7 (15.5) years, and 87 were men (40.7%) of their studied cases (8), due to more liability for viral exposure of males rather than females, as the biological differences in the immune systems between men and women exist which may impact our ability to fight an infection including SARS-2-CoV-2. Generally, females are more resistant to infections and this is possibly mediated by several factors including sex hormones and high expression of coronavirus receptors (ACE 2) in men, also life style, such as higher levels of smoking and drinking among men as compared to women. Additionally, women have more responsible attitude toward the Covid-19 pandemic than men. This may reversibly affect the undertaking of preventive measures such as frequent hand washing, wearing of face mask, and stay at home orders.

In this study, 24 (20.3%) patients had an acute ischemic stroke, and among them, 6 (9.5%) were non-haemorrhagic while 18 (32.7%) were haemorrhagic infarctions. This findings agreed with Meppiel et al. [6] who found 57 (25.6%) out of 222 patients had an acute ischaemic stroke.

Several mechanisms are likely to be associated. It is known that SARS-CoV-2 are associated with an increase of prothrombotic events such as ischemic stroke [7]. Thereby, viral infections may elevate procoagulant markers, leading to thrombosis, disseminated intravascular coagulation and haemorrhagic events [8]. It is also known that virus could directly lead to myocardial injury, promoting cardiac arrhythmias associated with embolic events. In the same way, a severe acute myocardial injury may be associated with a decrease in brain perfusion and therefore with watershed cerebral infarctions [9].

D-dimer, a fibrin-degraded product that serves as a marker of defective activation of the coagulation system, is often elevated in critically ill patients with extreme SARS-CoV-2 infection (with a mean level of 2.9 mg/L), that serves as a marker of dysfunctional activation of the coagulation system, such as in acute ischemic stroke. As Beyrouti et al. [10] recently mentioned, all our patients tested showed elevated D-dimers, markedly elevated (> 1.8 mg/L) for 10 of the 11 patients tested.

There are a few possible causes for the fifty-five cases of COVID-19-related intracerebral haemorrhage. In patients with hypertension, the expression and ability of the ACE2 receptor to lower blood pressure is known to be diminished [11]. The presence of S protein in SARS-CoV-2 infection can also reduce the expression and function of ACE2 proteins. Infected patients may experience uncontrolled hypertension, arterial wall rupture, and cerebral haemorrhage as a result of this [12]. Furthermore, if the virus disseminates in the cerebral microvasculature, damage to capillary endothelial cells and could cause a tear in the vasculature, resulting in parenchymal haemorrhage [13]. Thrombocytopenia and coagulopathy have also been identified in COVID-19 patients, both of which are risk factors for secondary brain parenchymal haemorrhage [14].

The present study discovered that altered sensorium and encephalopathy were common in COVID-19 patients. Cerebral edema is the most common pathological change seen in this condition, with headache, confusion, delirium, loss of consciousness, seizure, and coma as main clinical features [15]. COVID19 patients with altered sensorium should be identified as soon as possible, as there may be underlying reversible causes, such as impending respiratory failure that require timely intervention [16]. The pathophysiology behind the cerebral dysfunction is hypothesized to be in part inflammatory-mediated [17]. This is supported by the fact that the encephalopathic Italian patient had a dramatic response to high-dose steroids [18].

Acute encephalitis is also a clinical entity that may be detected in patients with COVID-19 infection. It is known that an infectious respiratory pathogen can infiltrate the CNS and lead to brain inflammation and degeneration of neurons [15]. In the current study (1.6%) had extensive cerebral involvement with acute haemorrhagic necrotizing leukoencephalitis.

After presenting with a 6-day history of cough, fever and altered mental status. That was coincided with El-Shourbagy et al. [19] who reported one case of encephalitis, that was associated with severe grade of pulmonary affection with COVID-19.

According to our knowledge, little publications had reported the MR spectroscopic results in infected patients with COVID 19 who had CNS complications. In the current study metabolic ratios were evaluated in 63 patients. It was observed that metabolic changes were as follows; elevated levels of choline, elevated Lac, decreased NAA, increased mI, and increased Glx ratios in relation to Cr. MRS findings were non-specific apart from marked increased Cho/Cr, decreased NAA/Cr, elevated Lac/Cr ratios, and more significant changes in severe cases of necrotizing leukoencephalopathy, prominent lactate peaks in ischemic areas.

Less pronounced changes in Cho/Cr and NAA/Cr ratios were noted in the patient with less severe MRI changes as demyelinating patches.

Long-term hypoxia in the white matter is thought to exacerbate anaerobic metabolism and result in elevated tissue lactate levels. Cho/Cr ratios have been found to increase in patients with leukoencephalopathy, especially in COVID-associated necrotizing leukoencephalopathy (owing to viral induced demyelinating process).

Cho elevation was related to cell death and/or immune cellular infiltration. Axonal damage was also reported on pathology and likely contributes to the decreased NAA/Cr ratios we observed. Increased levels of Myo-Inositol (mI) may reflect inflammation of neuronal cells. Glx levels have been shown to be elevated in cases of acute viral induced leukoencephalopathy, which was in line with Zawadzki et al. [20] where patients with extensive white matter abnormalities showed increased Cho/Cr, decreased NAA/Cr ratios, while the control had a relatively normal range of Cho/Cr ratios.

### Limitations of the study

The short duration cross-sectional nature of this study limits the ability to generalize our observations. MRS was only performed in patients when requested by referring providers for specific clinical indications. A single time point of assessment was also another limiting factor in this study.

## Conclusions

Finally, to conclude that MRI and MRS are non-invasive modalities to assess and evaluate COVID-19 induced neurological complications. MRS findings were non-specific apart from decreased NAA/Cr, elevated lactate peaks, elevated Lac/Cr ratios, and more significant changes in severe cases of encephalitis, prominent lactate peaks in ischemic areas. Moreover, MRS couldn’t be performed in haemorrhagic cases as blood component interfere with field proper shimming and homogeneity and misleads the MRS results.

## Recommendations

Continued data collection in a larger sample, longer duration with the ability of multi-points time of assessment is highly recommended to validate these observations and better elucidate their significance in the pathophysiology of COVID-19 induced CNS lesions. Multi-centric research studies in different countries all over the world are recommended in the future research field for assessment of the effect of different strains and serotypes of COVID-19 virus.

## Data Availability

The authors confirm that all data supporting the finding of the study are available within the article and the raw data supporting the findings were generated and available at the corresponding author on request.

## Abbreviations

COVID-19: Coronavirus disease 2019
FLAIR: Fluid-attenuated inversion recovery
SARS-CoV-2: Severe acute respiratory syndrome coronavirus 2
WM: White matter
MRI: Magnetic resonance imaging
MRS: Magnetic resonance spectroscopy
NAA: *N*-Acetyl aspartate
Cr: Creatine
Cho: Choline
mI: Myo-inositol
Glx: Glutamine and glutamate
Lac: Lactate
SD: Standard deviation
